# Neonatal sevoflurane anesthesia can also affect rat medulla spinalis

**DOI:** 10.2478/abm-2025-0017

**Published:** 2025-06-30

**Authors:** Elvan Ocmen, Hale Aksu Erdost, Osman Yilmaz, Alper Bagriyanik, Muge Kiray, Necati Gokmen

**Affiliations:** Department of Anesthesia, Dokuz Eylul University Hospital, Izmir 35330, Turkey; Department of Anesthesia, Dokuz Eylul University School of Health Sciences, Izmir 35330, Turkey; Department of Histology and Embryology, Dokuz Eylul University Hospital, Izmir 35330, Turkey; Department of Physiology, Dokuz Eylul University, Izmir 35330, Turkey

**Keywords:** animals, neonatal anesthesia, newborn, sevoflurane, spinal cord

## Abstract

**Background:**

Anesthesia has been linked to neuroapoptosis and prolonged neurocognitive disorders in the neonatal rat brain, but the full extent of damage induced by anesthesia on the central nervous system is still unknown.

**Objectives:**

We aim to investigate whether sevoflurane anesthesia affects the spinal cord.

**Methods:**

After the approval of the ethics committee, 24 Wistar albino rat pups, weighing between 9 g and 11 g, on the postnatal 7th day were included in the study. In the sevoflurane groups, rats breathed 2.5% sevoflurane in oxygen. The tail flick tests were performed on postnatal 8th, 15th, and 30th days to evaluate motor functions. At the end of the experiments, rats were sacrificed by decapitation, and their spinal cords were taken for histopathological evaluation.

**Results:**

There was a significant difference between the tail pulling times on the 8th and 30th days in both groups (*P* = 0.036). No significant difference was found between the control and sevoflurane groups (*P* = 0.053). In histopathological assessments, the chronic sevoflurane group showed a significant increase in apoptotic cell count (*P* < 0.001).

**Conclusions:**

This study showed that although there was a significant increase in apoptotic cells in the chronic sevoflurane group, motor function of the spinal cord was not affected. Further studies can be conducted to investigate the possible mechanisms.

Volatile anesthetics have been used safely for decades. However, the US Food and Drug Administration (FDA) issued a safety announcement in 2016 regarding the use of anesthesia in children and pregnant women after some studies showed that anesthesia could cause neurotoxicity and learning and memory defects. Sevoflurane is a volatile anesthetic used in pediatric patients for induction and maintenance. Many experimental studies have found that molecular and pathological changes arise in the developing brain after sevoflurane exposure. The results of clinical studies on anesthesia-induced neurotoxicity are controversial [1].

The exact mechanism of sevoflurane-induced neurotoxicity is unknown. Although several possible mechanisms have been suggested, including autophagy, apoptosis, micro ribonucleic acid (miRNAs), long non-coding RNA (lncRNAs), and neuroinflammation [1], the precise mechanism remains unclear. Sevoflurane targets the central nervous system, the depression of which results in anesthesia [2]. Therefore, it is not surprising that sevoflurane’s side effects affect the central nervous system as well.

Since learning and memory impairment occur after hippocampal injury, the brain, especially the hippocampus, has been studied mostly in anesthesia-induced neurotoxicity [1, 3]. However, it is known that other regions of the brain can also be affected by anesthesia [4]. Medulla spinalis, a part of the central nervous system, may also be damaged after inhalational anesthesia. Previously, Sanders et al. [5] showed that a combination of nitrous oxide with isoflurane can cause spinal apoptosis in neonatal rats.

The brain growth spurt is the rapid growth of the central nervous system. During this period, children are more vulnerable to neurotoxins. It has been shown that neurotoxins can also affect the spinal cord during the brain growth spurt [6]. This study evaluated the effects of a sole volatile anesthetic, sevoflurane, on the medulla spinalis in neonatal rats.

## Methods

After the approval of the Animal Research Committee of Dokuz Eylul University (52/2013), 24 Wistar albino rat pups (7-day-old) weighing 9‒11 g were involved in the study. Animals were obtained from Dokuz Eylul University Laboratory Animals Division and were kept in the same laboratory until the end of the experiments. They were housed under standard conditions (12 h day, 12 h night, 20°C‒22°C, and 50%‒60% humidity). All efforts were made to reduce the number of animals and their suffering.

Littermate pups were randomized into 4 groups by simple randomization:

- Control group acute (n = 6): Rats breathed 100% oxygen for 6 h. At the end of 6 h, rats were anesthetized by sevoflurane and decapitated; their spinal cords were taken for histopathological assessment.
- Control group chronic (n = 6): Rats breathed 100% oxygen for 6 h. The tail flick test was applied on postnatal 8th, 15th, and 30th days to evaluate motor functions. After the tests were completed, the rats were anesthetized with sevoflurane and sacrificed with decapitation. Their spinal cords were taken for histopathological assessment.
- Acute sevoflurane group (n = 6): Rats breathed 2.5% sevoflurane in 100% oxygen for 6 h [7]. After anesthesia, rats were sacrificed by decapitation. Their spinal cords were taken for histopathological assessment.
- Chronic sevoflurane group (n = 6): At the end of 2.5% sevoflurane anesthesia in 100% oxygen for 6 h, sevoflurane was discontinued and 100% O_2_ was inhaled to ensure their recovery. The tail flick test was performed on postnatal 8th, 15th, and 30th days to evaluate motor functions. After the tests were completed, the rats were anesthetized with sevoflurane and sacrificed with decapitation. Their spinal cords were taken for histopathological assessment.

To prevent unnecessary animal use, the “E” value was calculated (total number of animals − total number of groups). In the chronic groups, the animals that were suffering any pain or could not feed were scheduled to be euthanized using high concentrations of sevoflurane anesthesia.

### Anesthesia

Each rat was anesthetized in a separate glass jar as described in our previous study [4]. Inspired oxygen and sevoflurane concentrations were monitored (Anesthesia Gas Monitoring 1304; Br€uel & KjaerSound & Vibration Measurement A/S Naerum) throughout the anesthesia period.

### Histological evaluation

For light microscopic assessment, spinal cord samples were fixed in a 10% buffered formalin solution, processed by routine histological methods, and embedded in paraffin blocks. Serial sections of 5 μm thickness were taken from the paraffin blocks with a microtome (Thermo Finesse ME+). All sections were stained with cresyl violet. For cresyl violet staining, sections were stained with 0.1% cresyl violet for 20 min, differentiated by immersion in 96% ethanol, and cleared by immersion in xylene. The paraffin sections were stained to demonstrate apoptotic cells using the terminal deoxynucleotidyl transferase-mediated dUTP-biotin nick end labeling (TUNEL) method. TUNEL assay was performed using the in situ cell death detection kit (Roche) according to the manufacturer’s instructions. The TUNEL-positive cells were analyzed using an image analysis system (CellSens Entry 1.7, Olympus) consisting of a microscope (Olympus CX-41) and a video camera (Olympus DP25). The number of apoptotic cells was counted in 10 fields under 40× magnification, and the apoptotic index (percentage of apoptotic nuclei) was calculated as apoptotic nuclei/total nuclei counted × 100%. All counting procedures were performed blindly.

### Tail flick test

The tail flick test was performed with a tail flick device using radiant heat (TF 0703 Tail Flick, May, Commat Ltd. Sti). The day before anesthesia, all pups were placed on the tail flick device without measuring, and a learning exercise was performed. The actual tests were done on the postnatal 8th day (the day after anesthesia). Thermal stimulation was applied to a distal third cm of the tail. The time between the start of stimulus and movement of the tail was recorded as the tail flick time. The maximum response time (cut-off time) was set to 14.9 s to prevent the tail from being injured.

### Statistical analysis

Values were presented as median (min–max) and mean ± tandard error of the mean (SEM). Data analyses were carried out using SPSS version 24.0 (IBM Inc.,) for Windows software. Differences between the groups were evaluated using a one-way analysis of variance (ANOVA) post hoc Bonferroni test. The values of *P* < 0.05 were considered statistically significant.

## Results

Twenty-four rats were included in the study. All rat pups completed the experiment, but there was a problem in fixing 2 rats’ spinal cords in the chronic sevoflurane group so 2 more rats were added. Their tail-flick times were also used in the analysis.

In the control group, the tail flick time was 9.40 (7‒14.9) s (median [min–max]) on the 8th day. On the 30th day, the tail flick time was 9.30 (6.3‒13.7) s for the same group. The tail flick times of the sevoflurane group were 9.40 (4.2‒14.9) s and 5.95 (3.8–10.2) s on the 8th and 30th days, respectively. There was a significant difference between the tail flick times on the 8th and 30th days in both groups (*P* = 0.036). No significant difference was found between the control and sevoflurane groups (*P* = 0.053) (**Table 1**).

**Table 1.** Results of 8th, 15th, and 30th days tail flick tests of control and sevoflurane groups

	**8th day**	**15th day**	**30th day**
Control group	9.40 (7–14.9)	10.70 (7.7–14.9)	9.30 (6.3–13.7)*
Sevoflurane group	9.40 (4.2–14.9)	11.35 (6.0–14.9)	5.95 (3.8–10.2)*

Results are given as median (min–max).

* *P* < 0.05 when compared with 8th day results.

**Figure 1.**
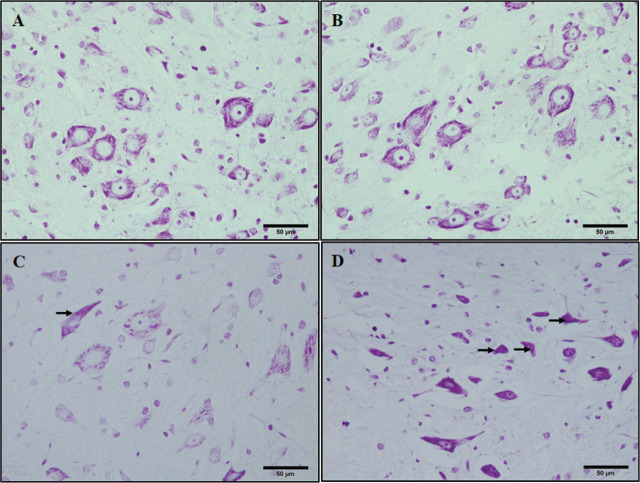
Crystal violet-stained sections of the rat spinal cords. **(A)** acute control, **(B)** chronic control, **(C)** acute sevoflurane, and **(D)** chronic sevoflurane groups. Arrows showing pyknotic cells. Bars = 50 μm.

In the histological evaluation, few pyknotic dark cells were found in the acute sevoflurane group whereas more degenerating dark cells were found in the chronic sevoflurane group in cresyl-violet stained images. In control groups, we did not observe any pyknotic dark cells (**Figure 1**).

**Figure 2.**
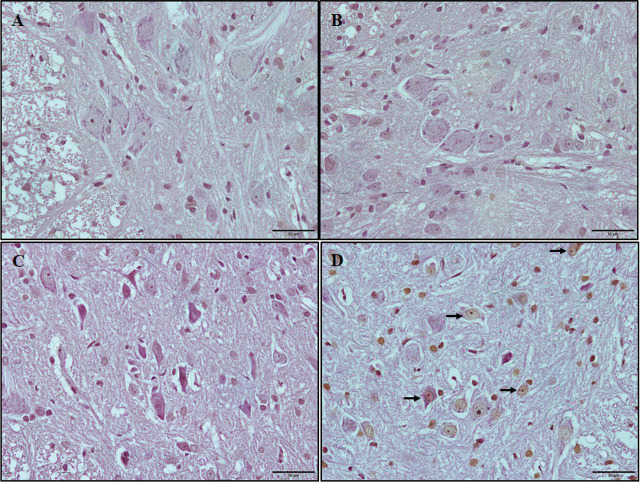
TUNEL staining. Representative photomicrographs of TUNEL-positive cells (arrows). **(A)** acute control, **(B)** chronic control, **(C)** acute sevoflurane, and **(D)** chronic sevoflurane groups. Bars = 50 μm.

TUNEL staining showed fewer TUNEL-positive cells in the control and acute sevoflurane groups than in the chronic sevoflurane group. There was no significant difference between the acute control, acute sevoflurane, and chronic control groups in the percentage of TUNEL-positive cells (*P* > 0.05). However, the number of TUNEL-positive cells increased significantly (3.24 ± 20.64, *P* < 0.05) in the chronic sevoflurane group (19.14 ± 2.81) compared with acute control (*P* < 0.001), acute sevoflurane (*P* = 0.001), and chronic control (*P* < 0.001) groups. Although there was an increase in TUNEL-positive cells in the acute sevoflurane group (9.85 ± 0.50) compared with the acute control group (4.28 ± 0.68), there was no statistically significant difference between groups (**Table 2**and **Figure 2**).

**Table 2.** The percentage of TUNEL-positive cells of ACont, ASevo, CCont, and CSevo groups

**Groups**	**n**	**TUNEL (%) (Mean ± SEM)**
ACont	6	4.28 ± 0.68
ASevo	6	9.85 ± 0.50
CCont	6	5.00 ± 0.65
CSevo	6	19.14 ± 2.81†
*P* (ACont vs. CSevo)		<0.001*
*P* (ASevo vs. CSevo)		0.001*
*P* (CCont vs. CSevo)		<0.001*

†* *P* < 0.05 compared with other groups.

ACont, acute control; ASevo, acute sevoflurane; CCont, chronic control; CSevo, chronic sevoflurane; SEM, standard error of the mean.

## Discussion

In this study, we demonstrated that sevoflurane anesthesia in 100% oxygen could cause apoptosis in the spinal cord in the late period, as shown by TUNEL and cresyl-violet staining. Although this finding did not affect the nociceptive function, the results show that anesthesia-related neurotoxicity may have a broader distribution in the central nervous system.

Anesthesia-related neurotoxicity has been a big concern since the beginning of the 2000s [8]. Many experimental studies show neuronal apoptosis or learning and memory deficits after anesthesia [9]. Although clinical studies have conflicting results, the FDA approved label changes for the use of general anesthetics in children in 2017 (https://www.fda.gov/drugs/drug-safety-and-availability/fda-drug-safety-communication-fda-approves-label-changes-use-general-anesthetic-and-sedation-drugs). Many anesthetic and sedative agents have been investigated for their neurotoxic effects. We chose sevoflurane because it is one of the most commonly used inhalational anesthetics in pediatric anesthesia. Possible mechanisms of anesthetic-related neurotoxicity are still being investigated. However, it is shown that *N*-methyl-d-aspartic acid (NMDA) and GABA type A (GABA_A_) receptors are involved in sevoflurane-induced neurotoxicity. During the brain growth spurt, drugs that affect these receptors, such as sevoflurane, may trigger widespread neurodegeneration. Sevoflurane-induced neurotoxicity is mainly associated with the duration and frequency of anesthesia and the age of the patient [1]. Newborns and infants have the highest risk for sevoflurane-induced neurodegeneration. Therefore, we designed a study to mimic prolonged sevoflurane anesthesia in newborns.

The spread of neuronal injury is also unclear [10]. Sanders et al. [5] showed that isoflurane anesthesia with nitrous oxide can cause apoptosis in the spinal cord of rat pups. The spinal cord is one of the least studied central nervous system sections in anesthesia-related neurotoxicity. Therefore, we decided to investigate the possible effects of sevoflurane anesthesia on the spinal cord. Moreover, this study showed that sevoflurane could cause apoptosis, non-significantly in the acute but significantly in the chronic group. Sanders et al. [5] found significant apoptosis in the spinal cord after the isoflurane and N2O combination but did not perform a histopathological examination after motor and nociceptive assessment. It is a well-known fact that anesthesia can cause learning and memory deficits; this indicates that the hazardous effects of anesthetics proceed over chronic periods. Isoflurane and N_2_O may have addictive, toxic effects on neurons. We used a sole anesthetic agent during the experiments and wanted to observe its effects. This could be the reason that we could not detect significant apoptosis after sevoflurane anesthesia.

The terminal deoxynucleotidyl transferase-mediated dUTP-biotin nick end labeling (TUNEL) method has been detecting apoptosis since 1992. The TUNEL method detects DNA fragmentation during apoptosis. It has been widely used for neurotoxicity studies for the spinal cord [11,12,13]. Cresyl-violet staining is also being used to detect neuronal cell death. Therefore, we used TUNEL and cresyl-violet staining to investigate the possible apoptotic effect of sevoflurane anesthesia on the spinal cord. We showed that sevoflurane could cause significant apoptosis 30 days after anesthesia.

The tail flick times on the 30th day were significantly shorter than the first day of the experiment in this study, both in the control and sevoflurane groups. This finding is probably associated with the rat pups’ learning period. Li et al. [14] investigated the effects of early isoflurane exposure on chronic pain pathways. They also used the tail flick test as one of their behavioral tests, 50 days after isoflurane exposure at P7. Unlike our findings, they found that the tail flick time was significantly shorter than the control group. They also showed that mTOR pathway activity and neuronal activity, which are associated with chronic pain, were increased in the insular cortex, anterior cingulate cortex, and spinal dorsal horn. They believe that isoflurane alters the mTOR pathway and increases the risk of persistent postsurgical pain. We performed the tail flick tests on P8, P15, and P30. Kang et al. previously showed that the critical time for mTOR activity is between P21 and P35 [15], so in their study setting, the timing of behavioral tests was much later than our tail flick test. Although we found significant apoptosis in the chronic sevoflurane group, we did not find any significant change in noxious stimuli during tail flick tests; mTOR activity may be the reason for our findings. Similar to our findings, Sanders et al. [5] could not detect any change in tail flick times after isoflurane anesthesia.

This is the first study investigating the effect of neonatal sevoflurane on the medulla spinalis. Learning and memory deficits and apoptosis in the brain after anesthesia exposure are widely investigated and still being studied, but medulla spinalis and motor function deficits studied by a few studies.

This study has some limitations. We used only one test (tail flick test) to evaluate the motor function of the rats after sevoflurane anesthesia. Unfortunately, we only have this test at our institute that can evaluate the motor function. We did not investigate the mechanisms that can induce apoptosis in the medulla spinalis after sevoflurane anesthesia. However, we aimed to reveal the possible neuroapoptosis in medulla spinalis, since, unlike apoptosis in the developing brain after anesthesia, the effect of sevoflurane on medulla spinalis is unknown. We used 100% oxygen. Oxygen has its own hazardous effects; however, this study aimed to evaluate the neurotoxic effects of sevoflurane. Although we could not find any difference between acute control and acute sevoflurane groups, we found a significant apoptosis with TUNEL staining in the chronic sevoflurane group. This finding may be explained by the scarification time of the chronic groups, as apoptosis may arise after 6 h. Since we did not find significant apoptosis in the chronic control group, the observed apoptotic effect is more likely to be associated with sevoflurane anesthesia.

In conclusion, we found that sevoflurane anesthesia in the early period of life could also be associated with neuronal cell death in the spinal cord without causing any alterations in the tail flick test. Our findings showed that anesthesia-related neurotoxicity may not be limited to the brain. The spinal cord could be a target of neonatal anesthesia. There are a few studies investigating medulla spinalis injury after general anesthesia. Further studies might consider studying the effects of early-life anesthesia on the medulla spinalis and investigating the possible interactions between neuroapoptosis and chronic pain pathways, such as mTOR activity.

## References

[1] Apai C, Shah R, Tran K, Pandya Shah S (2021). Anesthesia and the developing brain: a review of sevoflurane-induced neurotoxicity in pediatric populations. Clin Ther.

[2] Li X, Sun Y, Jin Q, Song D, Diao Y (2019). Kappa opioid receptor agonists improve postoperative cognitive dysfunction in rats via the JAK2/STAT3 signaling pathway. Int J Mol Med.

[3] Kang W, Lu D, Yang X, Ma W, Chen X, Chen K (2020). Sevoflurane induces hippocampal neuronal apoptosis by altering the level of neuropeptide Y in neonatal rats. Neurochem Res.

[4] Ocmen E, Derbent A, Micilli SC, Cankurt U, Aksu I, Dayi A (2016). Erythropoietin diminishes isoflurane-induced apoptosis in rat frontal cortex. Paediatr Anaesth.

[5] Sanders RD, Xu J, Shu Y, Fidalgo A, Ma D, Maze M (2008). General anesthetics induce apoptotic neurodegeneration in the neonatal rat spinal cord. Anesth Analg.

[6] Karlsson O, Lindquist NG, Brittebo EB, Roman E (2009). Selective brain uptake and behavioral effects of the cyanobacterial toxin BMAA (β-N-methylamino-L-alanine) following neonatal administration to rodents. Toxicol Sci.

[7] Wang S, Xue H, Xu Y, Niu J, Zhao P (2019). Sevoflurane postconditioning inhibits autophagy through activation of the extracellular signal-regulated kinase cascade, alleviating hypoxic-ischemic brain injury in neonatal rats. Neurochem Res.

[8] Jevtovic-Todorovic V, Hartman RE, Izumi Y, Benshoff ND, Dikranian K, Zorumski CF (2003). Early exposure to common anesthetic agents causes widespread neurodegeneration in the developing rat brain and persistent learning deficits. J Neurosci.

[9] Ing C, Warner DO, Sun LS, Flick RP, Davidson AJ, Vutskits L (2022). Anesthesia and developing brains: unanswered questions and proposed paths forward. Anesthesiology.

[10] Niu Y, Yan J, Jiang H (2022). Anesthesia and developing brain: what have we learned from recent studies. Front Mol Neurosci.

[11] Xue X, Lv Y, Leng Y, Zhang Y (2020). Autophagy activation attenuates the neurotoxicity of local anaesthetics by decreasing caspase-3 activity in rats. Braz J Anesthesiol.

[12] Sun G, Wang X, Li T, Qu S, Sun J (2018). Taurine attenuates acrylamide-induced apoptosis via a PI3K/AKT-dependent manner. Hum Exp Toxicol.

[13] Zheng X, Chen L, Du X, Cai J, Yu S, Wang H (2017). Effects of hyperbaric factors on lidocaine-induced apoptosis in spinal neurons and the role of p38 mitogen-activated protein kinase in rats with diabetic neuropathic pain. Exp Ther Med.

[14] Li Q, Mathena RP, Eregha ON, Mintz CD (2019). Effects of early exposure of isoflurane on chronic pain via the mammalian target of rapamycin signal pathway. Int J Mol Sci.

[15] Kang E, Jiang D, Ryu YK, Lim S, Kwak M, Gray CD (2017). Early postnatal exposure to isoflurane causes cognitive deficits and disrupts development of newborn hippocampal neurons via activation of the mTOR pathway. PLoS Biol.

